# Genetic parameters estimation for some wild wheat species and their F1 hybrids grown in different regions of Saudi Arabia

**DOI:** 10.1016/j.sjbs.2021.09.015

**Published:** 2021-09-15

**Authors:** Meshal M. Almutairi

**Affiliations:** National Center of Agricultural and Technology, King Abdulaziz City for Science and Technology (KACST), P.O. Box 6086, Riyadh 11442, Saudi Arabia

**Keywords:** Spring wheat (*Triticum aestivum*), Heterosis over better parent (HBP), Heterosis over mid parent (HMP), The genetic advance (GA), Genotype coefficient of variation (GCV), Phenotypic coefficient of variation (PCV)

## Abstract

Wheat (*Triticum aestivum* L.) is the most important crop for human nutrition that underpins the food safety of Saudi Arabia. The investigation here was to determine heterosis effects using different genetic methods: heterosis over better, mid parents, the genetic advance, and genotype, phenotypic coefficient of variation for estimation some traits among six wheat landraces and their F_1_ hybrids. In 2019, these landraces were sown using hand and after 100 days, the emasculation and crossing were made among these six landraces using hand emasculation of anthers. In 2020, seeds for these genotypes (six wheat landraces and their F_1_) were sown under normal irrigation accordingly done in 2019. The results showed that the most important parent was Mabia resulted with the highest value in number of tiller/ plant, 1,000-grain weight, and fresh shoot weight. The highest value of plant height among six parents was Naqra while highest value at the same trait among F_1_ hybrids was P_3_ XP_6_. The estimations of heterosis showed that out of 15 crosses, one cross (P_1_XP_5_) was significantly better yield than all crosses for these four traits. The genotype coefficient of variation (GCV) ranged from 12.5% to 8.7% while phenotypic coefficient of variation ranged from 17.7% to 11.3%. The correlation coefficients was found between fresh shoot weight and number of tiller and plant height and umber of tiller. Wild wheat still serve as a source of useful germplasm with proven adaption and productivity and thus assembles of the wild wheat assortments are the initial step of breeding program.

## 1 Introduction

Wheat (*Triticum aestivum* L., 2n = 6x = 42 = AABBDD genomes) is one of the most important crop in terms of grain production for human nutrition as the second cultivated crop (Ziegler et al., 2016, Paulsen and Shroyer, 2008). Wheat contains carbohydrate as a source of energy and has significant amount of important nutrition such as proteins, fiber, vitamins, and mineral (Shewry and Hey, 2015). In Kingdom of Saudi Arabia, the total wheat cultivated area was 897,000 m^2^, and total production was 530,000 tons in 2018 (General Authority for Statistics 2019). Local farmers depend on some wheat landraces for instance Al-Samma, Al-Qaima, Samira, and Madinah for both self-sufficient and local market purposes. These landraces grown in diverse regions of the country. The most cultivated areas are Al-Jouf, Qassim, and Riyadh represented a diversity of genetics background due to local adaption to diversity abiotic and biotic stresses (Sayed, 1979).

One of the main strategic objectives of Kingdom of Saudi Arabia is to achieve a sustainable system of the national food production through rising self-sufficient levels and improving productivity of agriculture (Saudi Grains Organization. 2019). This accomplishing of food system can be built through plant breeding based on genetic parameters among landraces. This demonstrates the necessity to classify wheat genetic gains for enabling breeders to exploit future genetic gains for selection and development local wheat. Plant breeders work hard for choosing the appropriate breeding methods to improve a desired trait and the main objective for breeders is to assess the general behavior of the genetic gains (Rivera-Amado et al., 2020, Zhang et al., 2017). Therefore, a developing wheat line is required for high potential yield as the main pillar of genetic improvement in wheat breeding regimes. Some studies were shown that the selection among hybrids could not play its active role unless there were genetic variations in the plant population resulting from all possible hybridizations that reflected adaption capacities (Mastrangelo and Cattivelli, 2021). Wheat landraces as genetic resources showed capacity for adaption to local environmental condition. In this concept, the sources of genetic diversity coming from landraces and synthetics of wheat have successfully practiced during pre-breeding program of wheat. These landraces of wheat can provide sources of increased grain weight and biomass under stress condition (Warburton et al., 2006, Zhang et al., 2017). The potential yield of wheat has reached an unchanged, which would result in decline yield regardless of significant works. Manipulation potential yield of wheat is a main goal for most of the breeders and thus exploitation of this yield through heterosis is important strategy to overcome the unchanged wheat yield (Rauf et al., 2012, Sharif et al., 2001). Today, consideration of best crop management strategies and development resistance species against biotic and abiotic stress, wheat needs more improvement for enhance genetic grains (Noorka and Afzal, 2009, Noorka and Haidery, 2011).

Heritability was described as the proportion of phenotypic variance, which comes from heritable genetic, designed as *h^2^* among individuals in a plant population. The main goal of estimating this heritability is to compare the expected gains from selection strategies under an experimental location. Plant breeders search the great diversity to estimate heritability among populations. Without this diversity of plant, the estimation of heritability complicates and in some case is not clear what the natural exact to be estimated, which would be lower the optimal breeding program and hard to breed a new variety (Nyquist, 1991). In addition. The advantage of heritability is recombination for adaption the target environment through phenotypical characteristic while the disadvantage is small number for examined loci of gene during morphology or pedigree analysis methods. This illustrates, therefore, a necessity to study the genetic variability of wheat in order to efficient development and management of local species by conservation and utilization in wheat breeding program. This heritability and advance genetic are clarify the nature of traits that can be developed through selections (Ahmed et al., 2007, Zaman et al., 2010, Zaman et al., 2011). Thus, this study has been designed to investigate genetic variability parameters in wheat landraces that grown under Saudi Arabia conditions.

This investigation was conducted to determine heterosis effects using different genetic methods such as heterosis over better parent, heterosis over mid parent, the genetic advance, and genotype coefficient of variation for estimation of some quantitative traits among six wheat landraces and their F_1_ hybrids to ascertain the future of breeding programs to great improvement of wheat yield.

## 2 Materials and methods

### 2.1 Plant materials

Seeds of Six wheat parents named Sama (P_1_), Najran (P_2_), Maeia (P_3_), Naqra (P_4_), Siyb (P_5_), and Mabia (P_6_) were collected from different geographical regions of Saudi Arabia (Riyadh, Al-Qasum and Najran, and Asir). This study was conducted in the experimental station of King Abualziz City for Science and Technology (KACST), Mizahmah city from 2019/2020 to 2020/2021. Samma and Naqra were from Riyadh region, Maeia was from Al-Qasum and Najran, Mabia was from Najran, and Siyb was from Asir.

### 2.2 Experimental design

These six parents were sown using hand during 25th November 2019 under normal condition. All parents and genotypes were irrigated daily, and the fertilization was done as normal recommended as agricultural practices.

Weeds, disease, and insect were controlled with recommended local herbicides. After 100 days and before flowering stage, the emasculation following by crossing were made among six parents by hand emasculation of anthers and bagging female of spikes then fertilization was done. The cross methods were half diallel cross as following: P_1_XP_2_, P_1_XP_3_, P_1_XP_4_, P_1_XP_5_, P_1_XP_6_, P_2_XP_3_, P_2_XP_4_, P_2_XP_5_, P_2_X P_6_, P_3_XP_4_, P_3_XP_5_, P_3_XP_6_, P_4_XP_5_, P_4_XP_6_, and P_5_XP_6_. The experimental plots consisted 4 rows, 2.5 m long and 5 cm between rows.

During 2020, seeds were collected from 15 randomly genotypes F_1_ with their parents and save them in separate bags. These seeds were sown in the next cropping season (November 2020 to end of March 2021) with three replications under normal irrigation with the fertilization accordingly done in 2019.

### 2.3 Statistical analysis and genetic parameters estimation

Data were analyzed with SAS program based on a randomized block design. The significant of difference of means was calculated by least significant difference (LSD). The estimation of heterosis was calculated according to Fonseca and Patterson (1968) as percentage of increase or decrease of F_1_ over both mid parent or better parent by following equation:

Heterosis over mid parent (HMP) (%) = F1-MP/MP*100

Heterosis over better parent (HBP) (%) = F1-BP/BP*100

Where, MP = mean mid parent, BP = mean better parent

The genetic advance (GA) was calculated according to formula reported by Johnson et al. (1989), Falconer, and Mackay (1989) alongside selection intensity of i = 2.06 for all traits as flowing:

$$\mathrm{GA}=h2*i*\sigma^{\left(2\right)}\mathrm{Ph}$$

where h^2^ heritability, i = 2.06, $\sigma^{2}ph$ phenotypic variance.

$h^{2}$was calculated according to Falconer, and Mackay (1989) by using formula adopted by Burton and Devane (1953) as following :

$h^{2}=$$\frac{\sigma^{2}G}{\sigma^{2}Ph}$ , where: $\sigma^{2}g\frac{\mathrm{MSG}-MSE}{r}$ is genotypic variance,$\sigma^{2}ph=\sigma^{2}g+\sigma^{2}e$,

σ^2^e = MSE according to Comstock and Robinson (1952) The mean values were used to calculate genotype coefficient of variation (GCV) and phenotypic coefficient of variation as described by Johnson et al. (1955) by using following formulas:

$$GCV\left(\%\right)=\frac{\sqrt{\sigma^{2}g}}{X}*100$$

$$PCV\left(\%\right)=\frac{\sqrt{\sigma^{2}ph}}{X}*100$$

Where: σ^2^g = genotypic variance, σ^2^ph = phenotypic variance, X  = sample mean.

## 3 Results

### 3.1 Analysis of variance

The means values and standard deviation for F_1_ hybrid and their parent are given in Table 1. Four agro-morphological traits named plant height, number of tillers/ plant, 1,000-grain weight, and fresh shoot weight showed significant differences at (P ≤ 0.01). The highest value of plant height among six parents was Naqra at 65.6 cm while highest value at the same traits among F_1_ hybrids was P_3_ XP_6_ at 72.7 cm. The most important parent was Mabia resulted with highest value in number of tillers/ plant, 1,000- grain weight and fresh shoot weight. However, Mabia showed low value in these three traits among F_1_ hybrids. These results indicated that both Naqra and Mbia needed more improvement through increasing the frequency of desirable alleles, which can be utilized the superior parents [24]. These results also indicated that all parents with their F_1_ hybrids prevalence of genetic variability.

**Table 1:** Mean values and standard deviation for evaluating F_1_ hybrid and their parent of wheat genotypes for four traits named plant height (PH), number of tiller/ plant (T/P), 1,000-grain weight (GW), and fresh shoot weight (FSW).

Genotypes	PH (cm)	T/P	GW (gm)	FSW (gm)
Samma (P_1_)	50.6 ± 1.72 efg	10.9 ± 1.43 cdef	29.3 ± 3.28 de	3.6 ± 0.41 de
Najran (P_2_)	64.1 ± 2.13 cd	10.6 ± 1.52 def	39.4 ± 4.66 abc	3.6 ± 0.50 cd
Maeia (P_3_)	46.4 ± 1.91 fg	13.0 ± 1.51 abcdef	33.7 ± 4.18 abcde	4.5 ± 0.49 abcde
Naqra (P_4_)	65.6 ± 3.02 bcd	11.1 ± 2.32 bcdef	33.0 ± 2.28 bcde	3.8 ± 0.93 bcde
Siyb (P_5_)	51.8 ± 1.69 fe	12.3 ± 1.37 abcdef	33.5 ± 0.45 bcde	4.2 ± 0.52 abcde
Mabia (P_6_)	43.1 ± 2.02 g	13.1 ± 1.33 abcdef	39.0 ± 3.96 abc	4.5 ± 0.66 abcde
P_1_ X P_2_	72.0 ± 2.82 ab	14.0 ± 0.84 abcd	32.2 ± 6.96 cde	5.0 ± 0.42 abcd
P_1_ X P_3_	65.7 ± 0.45 bcd	14.3 ± 1.03 abc	31.9 ± 1.02cde	5.1 ± 0.52 ab
P_1_ X P_4_	51.5 ± 0.63 fe	10.1 ± 0.98 ef	36.5 ± 4.25 abcd	3.5 ± 0.32 e
P_1_ X P_5_	52.9 ± 1.59 fe	13.8 ± 2.99 abcd	40.4 ± 3.51 ab	5.1 ± 1.24 abc
P_1_ X P_6_	52.1 ± 1.83 fe	13.1 ± 1.33 abcdef	33.1 ± 2.69 bcde	4.7 ± 0.88 abcde
P_2_ X P_3_	62.1 ± 6.20 cd	13.5 ± 1.22 abcde	29.0 ± 1.52 de	4.7 ± 0.61 abcde
P_2_ X P_4_	73.6 ± 2.73 a	12.1 ± 0.41 abcdef	28.5 ± 3.16 de	4.1 ± 0.20 abcde
P_2_ X P_5_	66.4 ± 5.41 abc	13.1 ± 1.72 abcdef	34.1 ± 2.11 abcde	4.8 ± 0.72 abcd
P_2_ X P_6_	58.1 ± 9.56 de	12.8 ± 2.32 abcdef	33.9 ± 2.13 abcde	4.4 ± 1.07abcde
P_3_ X P_4_	53.5 ± 1.63 ef	10.8 ± 1.33 cdef	35.1 ± 1.06 abcde	3.6 ± 0.41 cde
P_3_ X P_5_	62.8 ± 1.81 cd	14.5 ± 2.26 ab	35.8 ± 3.99 abcd	5.3 ± 0.93 a
P_3_ X P_6_	72.7 ± 3.50 ab	12.5 ± 0.49 abcdef	38.3 ± 6.08 abc	4.2 ± 0.27 abcde
P_4_ X P_5_	53.6 ± 1.96 ef	9.8 ± 1.71f	41.8 ± 2.48 a	3.5 ± 0.45 e
P_4_ X P_6_	67.5 ± 2.33 abc	15.0 ± 1.67 a	35.5 ± 8.14 abcd	5.4 ± 0.74 a
P_5_ X P_6_	72.0 ± 2.79 ab	11.3 ± 2.16 bcdef	27.1 ± 2.14 e	3.8 ± 0.93 bcde
C.V.	16.32 ± 9.78	17.21 ± 2.15	15.81 ± 5.42	20.67 ± 0.90
Std. deviation	9.65	2.21	5.24	0.89
Variance	93.19	4.52	27.46	0.80
LSD 0.01	3.90	1.92	4.35	0.81

Coefficient Variation (CV), least Significant Difference (LSD), values not sharing the same letter in a column differ significantly at 1% levels of probability.

#### 3.1.1 Analysis of heterosis over better parents

Data presenting in the Table 2 shows estimated heterosis for both mid parent (HMP) and better parent (HBP) for F_1_ hybrid wheat genotypes for four traits named plant height in cm (PH), number of tiller/ plant (T/P), 1,000-grain weight in gram (GW), and fresh shoot weight in gram (FSW. The results that all estimations were significantly different among hybrids for all traits. Out of 15 crosses, one cross (P_1_XP_5_) was significantly better yield than all crosses for these four traits. The hybrid means for the better parent ranged from positive to negative values. For the plant height, the positives and maximum heterosis from the largest to the smallest were in some genotypes named P_3_XP_6_, P_5_XP_6_, P_1_XP_3_, P_3_XP_5_, P_1_XP_2_, P_2_XP_4_, P_2_XP_5_, P_1_XP_6_, P_4_XP_5_, P_4_XP_6,_ and P1XP5. While the negative and minimum heterosis were P_1_XP_4_, P_2_XP_3_, P_2_X P_6_, P_3_XP_4_. For the number of tiller/ plant, the positives and maximum heterosis were in some genotypes named P_3_XP_6_, P_5_XP_6_, P_1_XP_3_, P_3_XP_5_, P_1_XP_2_, P_2_XP_4_, P_2_XP_5_, P_1_XP_6_, P_4_XP_5_, and P4XP6. However, the negative and minimum heterosis were P_1_XP_4_, P_1_XP_5_, P_2_XP_3_, P_2_X P_6_, and P_3_XP_4_. The combination between these two traits indicates that hybrid P_1_XP_2_, P_2_XP_4_, and P_2_XP_5_ were the most important hybrids. For the 1000- grain weight, the positives and maximum heterosis were P_4_XP_5_, P_1_XP_4_, P_1_XP_5_, P_3_XP_4_, and P_3_XP_5_, while the negative and minimum heterosis found in the reaming crosses. For the fresh shoot weight, the positive and maximum heterosis were P_1_XP_2_, P_4_XP_6_, P_1_XP_5_, P_3_XP_5_, P_2_XP_5_, P_1_XP_3_, P_1_XP_6_, P_2_X P_6_, P_2_XP_3_, and P_2_XP_4_, while the negative and minimum heterosis found in the reaming crosses.

**Table 2:** Estimates heterosis for both mid parent (HMP) and better parent (HBP) for F1 hybrid wheat genotypes for four traits named plant height in cm (PH), number of tiller/ plant (T/P), 1,000-grain weight in gram (GW), and fresh shoot weight in gram (FSW).

Genotypes	PH (cm)		T/P		GW		FSW	
	HMP*	HBP**	HMP	HBP	HMP	HBP	HMP	HBP
P1 X P2	−12.14	13.39	−13.85	28.24	−34.24	−18.18	−8.40	36.36
P1 X P3	−11.00	29.77	−17.70	10.26	−30.93	−5.43	−12.68	14.81
P1 X P4	−38.29	−21.47	−38.38	−8.96	−20.36	10.61	−37.31	−8.70
P1 X P5	−30.90	2.09	−19.02	12.16	−12.30	20.65	−12.23	19.61
P1 X P6	−27.80	2.96	−24.76	0.00	–32.25	−15.17	−20.28	3.64
P2 X P3	−28.78	−2.99	−21.36	3.85	−48.48	−26.43	−18.57	5.56
P2 X P4	–23.96	12.33	−25.13	8.96	−49.03	−27.70	−25.76	6.52
P2 X P5	−26.20	3.64	−21.78	6.76	−39.32	−13.53	−17.66	10.59
P2 X P6	–32.18	−9.34	−25.60	−2.53	−42.40	−13.91	–23.69	−2.18
P3 X P4	–32.35	−18.30	−41.70	−16.67	−29.98	4.25	−42.86	−18.52
P3 X P5	−13.13	21.22	−24.35	11.54	−29.93	4.84	−19.50	18.52
P3 X P6	6.99	56.73	−35.74	−3.21	−27.95	−1.62	−37.42	−5.56
P4 X P5	−41.44	3.38	−43.27	−20.27	−15.91	24.88	−41.26	−17.65
P4 X P6	–22.47	3.05	−15.49	13.92	–32.38	−8.97	−11.56	18.18
P5 X P6	−1.93	38.91	−40.09	−13.92	−48.74	−30.34	−41.40	−16.36

* HMP indicates Heterosis over mid parent, ** HBP indicates Heterosis over better parent.

#### 3.1.2 Analysis of heterosis over mid parents

The hybrid means for the mid parent had only negative values. For the plant height, the highest five values were in P_3_XP_6_, P_5_XP_6_, P_1_XP_3_, P_1_XP_2_, and P_3_XP_5_, while the other negative and minimum heterosis less than −20 found in the reaming crosses. For the number of tillers/ plant, the highest five values were in P_1_XP_2_, P_1_XP_3_, P_4_XP_6_, P_2_XP_5_, and P_1_XP_5_, while the other negative and minimum heterosis less than 20 found in the reaming crosses. For the 1000- grain weight, the highest values were in two crosses identified P_1_XP_5_ and P_4_XP_5_, while the other values in the reaming crosses. For the fresh shoot weight, the highest five values were in P_1_XP_2_, P_1_XP_3_, P_4_XP_6_, P_1_XP_5_, and P_2_XP_5_, while the other negative and minimum heterosis less than 20 found in the reaming crosses.

#### 3.1.3 Analysis of the genetic parameters

The genetic parameters as the genetic parameters, heritability, genotype and phenotypic coefficient of variation for plant height, number of tillers/ plant, 1,000- grain weight, and fresh shoot weight are shown in the Table 3.

**Table 3:** The genetic parameters for four traits.

Traits	σ^2^g	σ^2^ph	H^2^	GA	GCV%	PCV%
Plant height	46.20	56.18	0.82	94.4	12.5	11.3
number of tiller/ plant	1.35	3.43	0.39	2.74	9.3	14.8
1,000 grain weight	9.03	20.2	0.44	18.22	8.7	13.1
Fresh shoot weight	0.24	0.60	0.40	0.49	11.2	17.7

σ^2^g = genotypic variance, σ^2^ph = phenotypic variance, GA indicates the genetic advance, H^2^ signals of heritability, GCV% signals of genotype coefficient of variation.

The highest value of Heritability was in plant height with 0.97 (97%) while the lowest value was number of tillers/ plant with 0.75 (75%). The genetic advance was little difference as the highest value was in 1,000- grain weight (47.5) and the lowest value was in plant height (4.40). This heritability measures the extent of phenotype caused by action genes. The high value of genetic advance with high value of heritability found in 1,000- grain weight were 47.51 and 0.84 respectively. This indicates that that this trait, 1,000-grain weight, is important traits. The genotype and phenotypic coefficient of variation were calculated to explain variability in these four traits. The genotype coefficient of variation (GCV) ranged from 12.5% to 8.7% and phenotypic coefficient of variation ranged from 17.7% to 11.3%. The highest value of genotype coefficient of variation was in plant height whereas the highest value of phenotypic coefficient of variation was in fresh shoot weight. The Pearson correlation coefficients was calculated as in Table 4. The correlation was found between fresh shoot weight and number of tillers/ plant as 0.98 whereas the plant height and umber of tillers/ plant was correlated at 0.176, and the last positive correlated was found between plant heights and fresh shoot weight at 0.15. The negative correlation was found between 1000- grain weight and all three remaining traits.

**Table 4:** Pearson Correlation Coefficients among wheat traits.

Traits	Tiller number	1000 grain weight	Fresh shoot weight
Plant height	0.176 (0.05)	−0.2400 (0.005)	0.152 (0.09)
Tiller number		−0.047 (0.6)	0.982 (<0.0001)
1000 grain weight			−0.003 (0.98)

## 4 Discussion

Wheat (*Triticum aestivum* L.) is one of the most important cereal crops in terms of grain production for human nutrition (Ziegler et al., 2016, Paulsen and Shroyer, 2008). It is observed that a trait is governed by non-additive gene action reflects low genetic advance and low heritability while the trait governed by additive gene action reflects high in both genetic advance and heritability. The main objective of Kingdom of Saudi Arabia is to achieve a sustainable agricultural system through rising self-sufficiency levels and improving productivity of agriculture (Saudi Grains Organization. 2019). Plant breeders work hard for choosing the appropriate breeding methods to improve a desired trait in order to assess the general behavior of the genetic makeup (Zhang et al., 2017). Six wheat parents named Sama (P_1_), Najran (P_2_), Maeia (P_3_), Naqra (P_4_), Siyb (P_5_), and Mabia (P_6_) were selected due to their high genetic variations in order to determine heterosis effects using different genetic methods as mention above for estimation of some quantitative traits among F_1_ hybrids and their parents for ascertain the future of breeding programs. Johnson et al. (1955) suggested that comparing both heritability with genetic advance would be useful tools to more accurate predicting yield under phenotypic selection. The highest value of plant height among six parents was Naqra while highest value at the same traits among F_1_ hybrids was P_3_ XP_6_ at 72.7 cm while Mabia was the most important parent due to highest value in number of tillers/ plant, 1,000- grain weight, and fresh shoot weight. The heritability measures the extent of phenotype caused by action genes. The high value of genetic advance with high value of heritability found in 1,000- grain weight were 47.51 and 0.84 respectively. Reported by Sardana et al. (2007) noted that high heritability may not essentially to increase genetic gain unless existing sufficient genetics. However, low heritability with low genetics advance that was found in number of tillers/ plant indicates slow progress through selection. From the Table 3 that the heritability of 1000- grain weight was not high (0.44) and same thing with genetic advance (18.22) which had conflict result with several reports such as Eid. (2009). This conflict could be clarified as the different alleles and different loci that are expressed differently. The sustainable agricultural system depends on the sustainable development goals. In addition, the plant breeding is cornerstone for this sustainable supply not only for research and for development but also for highly specialized for improved traits in order to accelerate genetic gains (Małyska and Jacobi, 2018, Thudi et al., 2021, Almutairi, 2021).

## 5 Conclusion

The highest value of heritability was in plant height while the lowest value was observed in number of tillers/ plant with. The 1,000- grain weight showed high value of heritability with high genetic advance value; however, the yield and its component traits are governed by multigene that affected by environments (Ahmed et al. 2007). The highest value of plant height among six parents was Naqra at 65.6 cm while highest value at the same traits among F_1_ hybrids was in P_3_ XP_6_ at 72.7 cm. The most important parent was Mabia that it resulted with highest value in number of tillers/ plant, 1,000- grain weight, and fresh shoot weight; however, this Mabia showed low value among F_1_ hybrids and thus Naqra and Mbia needed more improvement through increasing the frequency of desirable alleles, which can be utilized the superior parents (Sleper and Poehlman, 2006). These two parents may need to be identified and increased as a pure population. Wild wheat still serve as a source of useful germplasm with proven adaption and productivity. Therefore, assembles of the wild wheat assortments are the initial step of breeding program that could contribute to improve wheat performance. The germplasm accessions should be grown initially in the local environment to identify sources of genes for maturity, disease resistance, and yield potential (Sleper and Poehlman, 2006). Saudi Arabia represents a diversity environmental condition and thus the wild wheat has cultivated in this diversity. Therefore, the results here were represented environment variability that concluded with fundamentally of the additive genes to possible yield potential development after several selection cycles (Sleper and Poehlman, 2006).

## Funding

This paper did not receive any funding resources or specific grant from agencies in the public, commercial, or any other sectors.

## Declaration of Competing Interest

The authors declare that they have no known competing financial interests or personal relationships that could have appeared to influence the work reported in this paper.
